# Edward Tobie, M. D.

**Published:** 1889-06

**Authors:** 


### EDWARD TOBIE, M. D.

AT A SPECIAL MEETING of the Medical Society of Erie County, held at the Y. M. C. A. building, Monday evening, May 13, 1889, the President, Dr. R. L. Banta, appointed the following-named members a committee to prepare a suitable memorial, expressive of the sorrow of the society upon the death of Dr. Tobie, viz.: Dr. J. Hauenstein, Dr. C. Diehl, Dr. Edward Storck, Dr. E. T. Dorland, and Dr. Thomas Lothrop. This committee subsequently reported as follows:

IN MEMORIAM.

EDWARD TOBIE, M. D.

Died May 12, 1889,

Aged 58 years.

In the death of the subject of this memorial, the medical profession loses an earnest, energetic, and esteemed member. His professional career of over thirty years, all of which was passed in this city, furnishes an example of toil and personal sacrifice in behalf of suffering humanity, with scarcely a parallel among his associates and collaborators. Devotion to professional duty, without regard to personal comfort or convenience or pecuniary gain, hastened the end to a life which with less of spirit of self-sacrifice would have been prolonged beyond the allotted of man.

Edward Tobie possessed the noblest qualities of head and heart. His superior mental gifts were improved with unremitting industry until he acquired a liberal education. His aptitude for linguistic studies enabled him to speak with fluency and grammatical accuracy several of the modern languages, that gave him the entrée to a large and influential patronage, to which he devoted all that he possessed of zeal and energy and skill.

Endowed by nature with marked independence of character, which were trammeled in his native land, he sought, at an early age, the freer political atmosphere of the western world, where he found a congenial field for the development of those manly qualities that subsequently commanded the high respect and affection of the community in which he lived.

In the medical profession, he acquired a wide reputation as a skilful physician, a close and accurate diagnostician, and a safe and reliable medical adviser. He possessed a warm and true heart and a sympathetic nature, which quickly responded to human suffering, and closely interwove his life in the important office of the physician, in the trials and sorrows of myriads of devoted friends and patients, whose grief for his sudden death attest the sincerity of their love and the gratitude they bore for the valuable and faithful service he rendered. To the poor, he demonstrated that the greatest of the blended virtues is charity, and the daily ministrations of kindness and mercy to the less fortunate of his fellow-men, were among the noble incidents of his busy life.

In the domestic circle, he exemplified the truest affection due to the companion of his life; and we beg the precious privilege to share in the sorrow which she feels in the sad event which robs her of the strength and support of a devoted husband.

In the fulness of his mature years, in the ripeness of a well-rounded life, with the respect of his brother physicians and fellow-citizens, with the deepest love and affection of his many patients, Edward Tobie has been summoned to a well-earned rest, and to those rewards merited through the conscientious performance of his life-work.

